# The strategic combination of trastuzumab emtansine with oncolytic rhabdoviruses leads to therapeutic synergy

**DOI:** 10.1038/s42003-020-0972-7

**Published:** 2020-05-22

**Authors:** Rozanne Arulanandam, Zaid Taha, Vanessa Garcia, Mohammed Selman, Andrew Chen, Oliver Varette, Anna Jirovec, Keara Sutherland, Elizabeth Macdonald, Fanny Tzelepis, Harsimrat Birdi, Nouf Alluqmani, Anne Landry, Anabel Bergeron, Barbara Vanderhyden, Jean-Simon Diallo

**Affiliations:** 1 Centre for Innovative Cancer Therapeutics, Ottawa Hospital Research Institute, Ottawa, ON K1H 8L6 Canada; 2 Department of Biochemistry, Microbiology, and Immunology, Faculty of Medicine, University of Ottawa, Ottawa, ON K1H 8M5 Canada; 3 Department of Cellular and Molecular Medicine, University of Ottawa, Ottawa, ON K1H 8M5 Canada

**Keywords:** Targeted therapies, Chemotherapy

## Abstract

We have demonstrated that microtubule destabilizing agents (MDAs) can sensitize tumors to oncolytic vesicular stomatitis virus (VSVΔ51) in various preclinical models of cancer. The clinically approved T-DM1 (Kadcyla®) is an antibody-drug conjugate consisting of HER2-targeting trastuzumab linked to the potent MDA and maytansine derivative DM1. We reveal that combining T-DM1 with VSVΔ51 leads to increased viral spread and tumor killing in trastuzumab-binding, VSVΔ51-resistant cancer cells. In vivo, co-treatment of VSVΔ51 and T-DM1 increased overall survival in HER2-overexpressing, but trastuzumab-refractory, JIMT1 human breast cancer xenografts compared to monotherapies. Furthermore, viral spread in cultured HER2^+^ human ovarian cancer patient-derived ascites samples was enhanced by the combination of VSVΔ51 and T-DM1. Our data using the clinically approved Kadcyla® in combination with VSVΔ51 demonstrates proof of concept that targeted delivery of a viral-sensitizing molecule using an antibody-drug conjugate can enhance oncolytic virus activity and provides rationale for translation of this approach.

## Abstract (web summary)

Arulanandam et al. demonstrate that HER2 positive cancer cells are sensitized to oncolytic rhabdovirus by an antibody-drug conjugate consisting of HER2- targeting trastuzumab linked to the microtubule destabiliser DM1. This study suggests the potential of such combination therapy in the treatment of HER2-positive cancers.

## Introduction

Human epidermal growth factor receptor-2 (HER2) is overexpressed in ~25–30% of breast cancers, but also in other malignancies including gastric cancer, head and neck cancer, and ovarian cancer^1–5^. Trastuzumab, a humanized IgG1-derived monoclonal antibody, binds to the extracellular domain IV of HER2. Trastuzumab (Herceptin®) is a mainstay for the treatment of HER2^+^ breast cancer and is also used in the context of gastric cancer^6–8^. However, only about 25% of HER2-overexpressing breast cancers respond to trastuzumab, in part due to therapeutic resistance^9^. The antibody–drug conjugate consisting of trastuzumab linked to the maytansine derivative DM1 (T-DM1 or Kadcyla®) can bypass HER2-signaling-associated resistance mechanisms through DM1-induced microtubule destabilization and cytotoxicity. As the first HER2-targeted antibody–drug conjugate (ADC) to be approved for clinical use, T-DM1 has shown some benefit in the treatment of HER2^+^ breast cancer patients that are refractory to trastuzumab alone^7,10^.

Oncolytic viruses have the potential to treat a broad range of malignancies and have recently made their way to the clinic, with the first such biotherapeutic approved for treatment of melanoma and many more in clinical development^11^. Yet, like most therapies, heterogeneity of treatment response to oncolytic viruses remains a major challenge. We have previously identified a number of small molecule viral sensitizers which are able to target the cellular antiviral response in multiple ways to potentiate the activity of oncolytic viruses in cancer cells in vitro and in vivo^12–16^. One such category of VSe molecules consists of microtubule destabilizers (MDAs) such as colchicine and vinorelbine, which specifically synergize with oncolytic rhabdoviruses including Vesicular Stomatitis Virus (VSV) and Maraba^12^. MDAs work by (1) improving viral spread in cancer cells through translational blocking of type I interferon (IFN) production and (2) enhancing cytokine-mediated polynucleation and bystander killing of surrounding, uninfected cancer cells. This was demonstrated in numerous cancer cell lines and mouse syngeneic or transgenic tumor models^12^. While effective, major limitations of this approach include systemic toxicity and a narrow therapeutic window inextricably linked to on-target effects of MDAs. To overcome these issues, we considered antibody–drug conjugate (ADC) technology as a means of focusing the delivery and accumulation of MDAs specifically to the tumor compartment. As proof of concept for this approach, we explored the use of the clinically approved Kadcyla® in order to sensitize HER2^+^ cancer cells to oncolytic VSV. In line with our published data using MDAs, we demonstrate in this study that combining T-DM1 with oncolytic VSV in vitro can increase viral infection and spread of cancer cells expressing HER2 with no impact on normal GM38 cells, while trastuzumab alone had no effect. We show that this tumor killing synergy is observed in VSVΔ51-resistant and HER2-low 786-0 renal carcinoma cells, as well as HER2-high breast and ovarian carcinoma lines. In vivo, co-treatment of VSVΔ51 and T-DM1 delays tumor progression in HER2-overexpressing, but trastuzumab-refractory, JIMT1 human breast cancer xenografts compared to monotherapies. Furthermore, viral spread in cultured HER2^+^ human ovarian cancer patient-derived ascites samples is also enhanced by the combination of VSVΔ51 and clinically relevant concentrations of T-DM1.

## Results

### T-DM1 improves VSVΔ51 oncolysis in resistant cancer cells

**Fig. 1:**
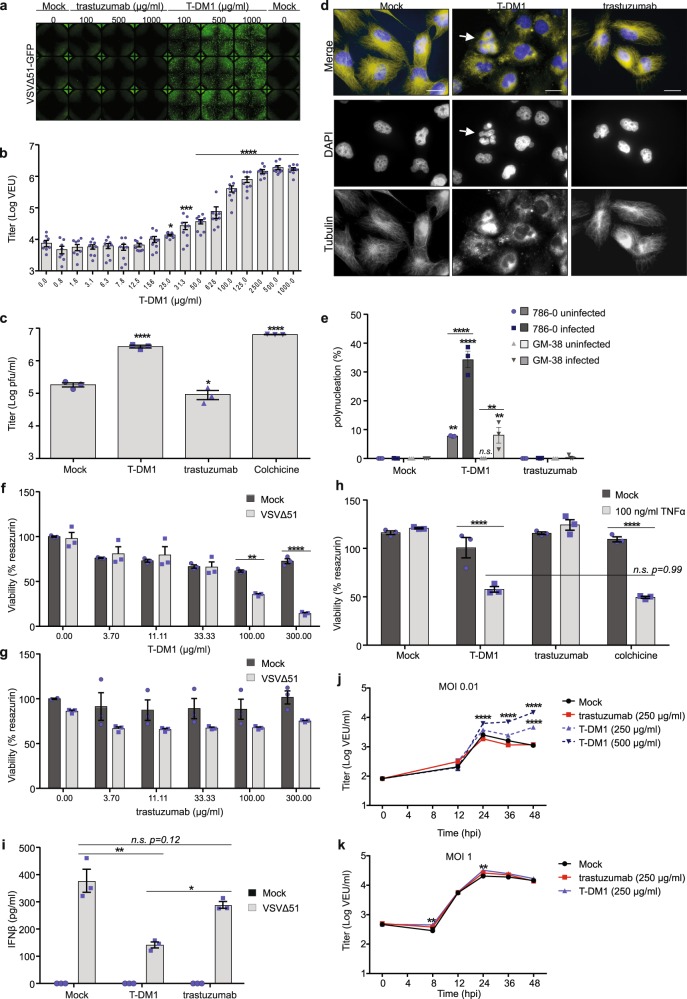
Conjugation of DM1 to trastuzumab increases VSVΔ51 spread and bystander killing in VSVΔ51-resistant cancer cells. **a** 786-0 cells were pretreated with T-DM1 or trastuzumab for 4 h followed by infection with VSVΔ51-GFP at MOI 0.01 and fluorescent images taken 24 h later, *n* = 3. **b** A high-throughput method^29^ was used to quantify viral titer from 786-0 pretreated with T-DM1 and infected with VSVΔ51-Fluc at MOI 0.1 (*n* = 9; mean ± SEM; one-way ANOVA compared to 0 µg/ml). **c** 786-0 were pretreated with either 100 μg/ml trastuzumab or T-DM1, 100 nM colchicine or Mock-treated, infected with VSVΔ51-GFP at MOI 0.01 and plaque assay performed after 48 h (*n* = 3; mean ± SEM; one-way ANOVA compared to Mock). **d** 786-0 were plated on glass coverslips, treated with 100 µg/ml T-DM1 or trastuzumab or mock, and then stained for β-tubulin and DAPI 24 h later. Objective = ×63, scale bar = 50 µm. White arrows indicate polynuclear cells. **e** Quantification of polynuclear 786-0 or GM38 cells from **d**. Images were taken 24 h post infection and 6 frames per experimental condition from *n* = 3 were scored for % polynuclear cells with SEM. Two-way ANOVA with Sidak’s multiple comparisons test was performed over mock or trastuzumab, or between groups indicated with bars, for each cell line. **f**, **g** 786-0 were treated as in **a**, and viability assessed 48 h post infection (*n* = 3; mean ± SEM; two-way ANOVA with Sidak’s multiple comparisons test across concentrations). **h** 786-0 treated as in **c** were administered 0 or 100 ng/ml TNFα and viability assessed after 72 h (*n* = 3; mean ± SEM); two-way ANOVA with Sidak’s multiple comparisons test across pretreatment conditions. **i** 786-0 were pretreated as in **a**, infected with VSVΔ51-GFP at MOI 0.1 or Mock. and supernatants assayed for IFNβ production 20 h later (*n* = 3; mean ± SEM; one-way ANOVA with Tukey’s multiple comparisons test across either Mock or VSVΔ51). **j**, **k** 786-0 were treated indicated and infected with VSVΔ51-Fluc at MOI 0.01 (**j**, *n* = 6) or MOI 1 (**k**, *n* = 3) and titered as in **b** (mean ± SEM; two-way ANOVA with Dunnett’s multiple comparison test compared to Mock for each time point).

Owing to their potent cytotoxicity, tubulin inhibitors have been a mainstay for cancer therapy and are the most validated payloads used for the design of ADC-mediated targeted therapies^17–19^. Conjugation of highly potent MDAs to antibodies can vastly improve their therapeutic window and tumor specificity^20,21^. Trastuzumab emtansine (T-DM1) allows delivery of the otherwise toxic DM1 (maytansine derivative) specifically to HER2-overexpressing cells^22–27^. T-DM1 has been clinically approved for the treatment of metastatic breast cancers that have previously been treated with trastuzumab and a taxane chemotherapy^7,28^. Given that microtubule inhibitors can potentiate cancer-specific spread and oncolysis mediated by VSVΔ51^12^, we sought to examine the combination of T-DM1 with VSVΔ51. As a starting point, we used VSVΔ51-resistant, interferon-responsive 786-0 renal carcinoma cells, a well-characterized system in our lab for assessing the impact of viral sensitizers^12,14,15^. 786-0 cells also express detectable levels of HER2, and are able to bind Trastuzumab (Supplementary Figs. 1 and 2). 786-0 cells were treated with increasing concentrations of T-DM1, and subsequently infected with VSVΔ51 encoding either GFP or luciferase, at a low multiplicity of infection (MOI 0.01–0.1). After 24 h, viral spread was followed by transgene expression using fluorescence microscopy to detect or quantify GFP (Fig. 1a and Supplementary Fig. 3). Viral titers were first estimated by comparing luciferase gene expression to that of a standard curve to compute viral expression units (VEU, Fig. 1b) as previously described^29^. A significant, dose dependent increase in VSVΔ51 output was observed with T-DM1 at concentrations >25 µg/ml and peaking at 500 µg/ml (Fig. 1b). This observation was confirmed by standard plaque assay, and the observed increase in viral titer was found to be significant over mock upon treatment of 786-0 cells with either T-DM1 or the control MDA colchicine, while trastuzumab alone had no positive impact (Fig. 1c). The boost in viral titers with T-DM1 also correlated with a disruption of the microtubule network in 786-0 cells as observed by immunofluorescence staining for β-tubulin and an increase in the number of polynuclear cells from 7% (with T-DM1 alone) to 33% (Fig. 1d, e). A significant enhancement in cytotoxicity measured by resazurin assay was also observed with T-DM1 doses of 100–300 µg/ml (Fig. 1f). In contrast, pretreatment of 786-0 cells with trastuzumab alone followed by VSVΔ51 infection did not impact microtubule depolymerization or cell death (Fig. 1d, e, g). Importantly, T-DM1 did not lead to similar levels of polynucleation, VSVΔ51 infection, or cell killing in normal GM38 fibroblasts (Fig. 1e, Supplementary Fig. 4) even though they express similar levels of HER2 and bind trastuzumab (Supplementary Figs. 1 and 2). Thus, taken together these data suggest that T-DM1-mediated sensitization to virus infection is cancer-specific and mediated by the MDA payload DM1, as opposed to trastuzumab alone.

We have previously demonstrated that MDAs, in addition to facilitating VSVΔ51 spread by blocking IFNβ secretion, can increase bystander cell killing by virus-induced cytokines such as TNFα^12^. In line with this, we found that T-DM1 increased cell death induced by TNFα in 786-0 cells by ~25%, to a similar degree as colchicine, whereas trastuzumab alone had no impact (Fig. 1h). We also confirmed that pretreatment of 786-0 cells with T-DM1 led to a significant reduction in secreted IFNβ levels 20 h post VSVΔ51 infection while trastuzumab treatment did not (Fig. 1i). To confirm that T-DM1 works by a similar mechanism to naked MDAs, a microarray analysis was conducted on mRNA obtained from cells pretreated with either T-DM1, colchicine, trastuzumab or mock-treated, prior to and following VSVΔ51 infection. Clustering analyses confirmed that the gene expression profile of T-DM1-treated 786-0 cells closely resembles that of colchicine in both infected and uninfected conditions, suggesting again that these drugs work by similar mechanisms (Supplementary Fig. 5a). Indeed, qRT-PCR analysis confirmed downregulation of the interferon stimulated gene known as interferon-induced transmembrane protein (IFITM1) following treatment with T-DM1 or colchicine, with no significant impact of trastuzumab alone (Supplementary Fig. 5b). Finally, comparing multi-step and single-step virus growth profiles suggested that in cancer cells, microtubule destabilization induced by T-DM1 has a more significant and direct impact on VSVΔ51 spread rather than replication (Fig. 1j, k), similar to what we observed with colchicine^12^. Overall, these data demonstrate that, like small molecule MDAs, T-DM1 can increase VSVΔ51 viral spread and oncolysis in VSV-resistant cancer cells by dampening the type I IFN pathway and increasing bystander killing.

### T-DM1 with VSVΔ51 increases oncolysis of HER2^+^ cancer cells

**Fig. 2:**
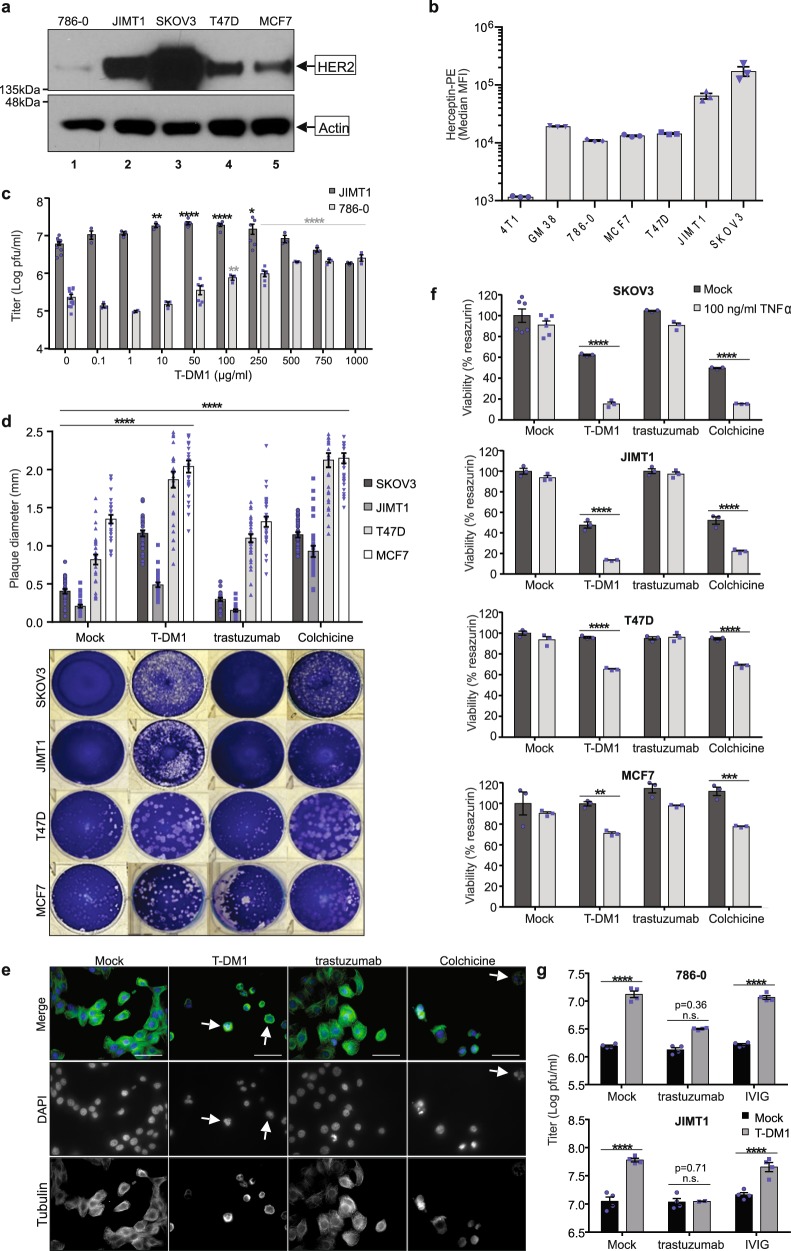
T-DM1 increases VSVΔ51 activity in a panel of HER2-expressing breast and ovarian cancer lines. **a** A panel of human breast and ovarian carcinoma lines were lysed and subject to western blotting for human HER2. **b** Mouse breast carcinoma 4T1 cells, and a panel of human cancer cell lines were subject to extracellular staining with Herceptin®, followed by goat anti-human IgG-PE. PE signal (*y* axis) was analyzed by flow cytometry, median MFI are shown; *n* = 3 ± SD. **c** 786-0 and JIMT1 cells were pretreated with T-DM1 for 4 h, washed and infected with VSVΔ51 (MOI 0.05 and 0.01, respectively) and plaque assay performed 45 h later (*n* = 3; mean ± SEM; one-way ANOVA with Dunnett’s multiple comparisons test compared to 0 µg/ml T-DM1 for each cell line). **d** Indicated cell lines were pretreated with either 100 nM colchicine, 100 μg/ml T-DM1 or trastuzumab, or mock, for 3 h, then washed and infected with VSVΔ51 (MOI 0.01 for JIMT1 and MOI 0.001 for others). 1 h later, wells were overlayed with agarose, and plaques visualized with Coomassie blue 48 h later. Diameter of 30 plaques per well were graphed (mean ± SEM; one-way ANOVA with Dunnett’s multiple comparisons test compared to Mock for each cell line). **e** JIMT1 cells were plated on glass coverslips, treated with 100 µg/ml T-DM1 or trastuzumab, or mock-treated, then stained for β-tubulin and DAPI 24 h later. Objective = ×63, scale bar = 50 µm, *n* = 3. White arrow indicates polynuclear cells. **f** Cell lines in **d** were pretreated as indicated then administered 0 or 100 ng/ml TNFα. Viability was determined 72 h later (*n* = 3; mean ± SEM; two-way ANOVA with Sidak’s multiple comparisons test for each pretreatment condition). **g** 786-0 or JIMT1 cells were either Mock-treated or treated with 1000 µg/ml T-DM1 (786-0) or 250 µg/ml T-DM1 (JIMT1) for 2 h, followed by an equivalent concentration of trastuzumab or IVIG. 2 h later, cells were infected with VSVΔ51 at MOI 0.01 and plaque assay performed 45 h later (*n* = 4; mean ± SEM; two-way ANOVA with Sidak’s multiple comparisons test compared to Mock for each cell line).

786-0 renal carcinoma cells offer a convenient model to study enhancers of VSVΔ51 given their inherent resistance to this virus. 786-0 cells display detectable HER2 levels as determined by western blotting (Fig. 2a, Supplementary Fig. 6), flow cytometry and immunofluorescence, when using trastuzumab as a primary antibody (Fig. 2b, Supplementary Figs. 1 and 2). HER2 is frequently amplified in breast and ovarian cancers, as well as other malignancies. In a given panel of these cell lines, Fig. 2a reveals that JIMT1 (breast) and SKOV3 (ovarian) express relatively high levels of HER2 while T47D (breast) and MCF7 (breast) express moderate to low HER2 levels. In comparison, 786-0 cells and normal GM38 cells display low levels of HER2 while 4T1 mouse breast carcinoma are essentially negative for this protein (Fig. 2a, b, Supplementary Figs. 1 and 2). To assess the potential of the T-DM1 combination with VSVΔ51 in HER2-overexpressing cancer cells, we pretreated JIMT1 cells with increasing concentrations of T-DM1 for 4 h followed by infection with VSVΔ51 at a low MOI. We used 786-0 as a HER2-low control for comparison and performed plaque assays 48 h later with infectious supernatants. Data reveal a significant increase in viral titers with lower amounts of T-DM1 (10–100 µg/ml) in HER2-high JIMT1 compared to HER2-low 786-0 cells, where 100–1000 µg/ml T-DM1 was required to enhance viral output (Fig. 2c). However, the fold enhancement in titer observed in 786-0 with T-DM1 (>100×) was greater than that observed in JIMT1 (~10×) due to the greater resistance of 786-0 cells to VSVΔ51 infection, with baseline titers being at least 100× lower in 786-0 compared to JIMT1 (Supplementary Fig. 7). To further examine the impact of T-DM1 on viral spread and oncolysis in our panel of HER2-expressing lines, JIMT1, SKOV3, T47D and MCF7 cells were seeded to confluency in 6-well dishes, and pretreated with T-DM1, colchicine, trastuzumab, or mock-treated for 3-4 h. This was followed by infection with VSVΔ51 at MOI 0.001 or 0.01, as indicated. After 1 h, an agarose overlay was added to the wells and cells were fixed after 24–48 h to quantify plaque diameter, as an indicator of viral spread and cell killing. Data reveal a ~2-fold increase in plaque size with T-DM1 or colchicine in HER2-expressing lines, whereas trastuzumab had no impact on VSVΔ51 spread (Fig. 2d). To confirm that T-DM1 is acting specifically through HER2, we tested its effects in 4T1.2 and 4T1.2 stably expressing erbB2/huHER2^30^. HER2 expression was confirmed in 4T1.2-HER2 cells by flow cytometry (Supplementary Figs. 2 and 8a) and immunofluorescence staining (Supplementary Fig. 8b) using trastuzumab as a primary antibody. Viral spread and oncolysis was next assessed as above revealing that while colchicine pretreatment could increase VSVΔ51 plaque area ~2× in both 4T1.2 and 4T1.2-HER2 cells, T-DM1 (100 µg/ml) was only able to significantly increase plaque size in 4T1.2-HER2 cells (Supplementary Fig. 8c, d). Importantly, microtubule disruption and polynucleation were observed in 4T1.2-HER2 following treatment with 100 µg/ml T-DM1 but not in HER2-negative 4T1.2 cells indicating a requirement for HER2 expression in T-DM1-mediated viral sensitization effects (Supplementary Fig. 8e). T-DM1 pretreatment of HER2-overexpressing JIMT1 cells also led to microtubule disruption, with a similar impact as colchicine (Fig. 2e). To further examine the impact of T-DM1 on cytokine-mediated bystander killing in our panel of breast and ovarian cancer cell lines, we treated JIMT1, SKOV3, T47D and MCF7 cells with T-DM1, colchicine, trastuzumab or vehicle, in the presence or absence of 100 µg/ml TNFα. Similar to our observations in 786-0 cells, T-DM1 or colchicine increased cell death induced by TNFα in these cells, with no significant impact of trastuzumab alone (Fig. 2f). Finally, to determine whether the viral enhancement observed was dependent on T-DM1 specifically, 786-0 or JIMT1 cells were pretreated for 2 h with either media alone, trastuzumab or a suspension of intravenous immunoglobulin (IVIG) as control, followed by an equivalent concentration of T-DM1 or media alone for a further 2 h. Cells were then washed and infected with VSVΔ51 at a low MOI and viral titers quantified by high-throughput assay. Data reveal that blocking HER2 binding sites through trastuzumab pretreatment abrogated the increase in viral titers induced by T-DM1 (Fig. 2g). Taken together, these data demonstrate that T-DM1 acts through HER2 and can lead to microtubule disruption and increased VSVΔ51-mediated oncolysis in HER2-expressing cancer cells.

### T-DM1 with VSVΔ51 improves survival of mice with HER2^+^ cancer

**Fig. 3:**
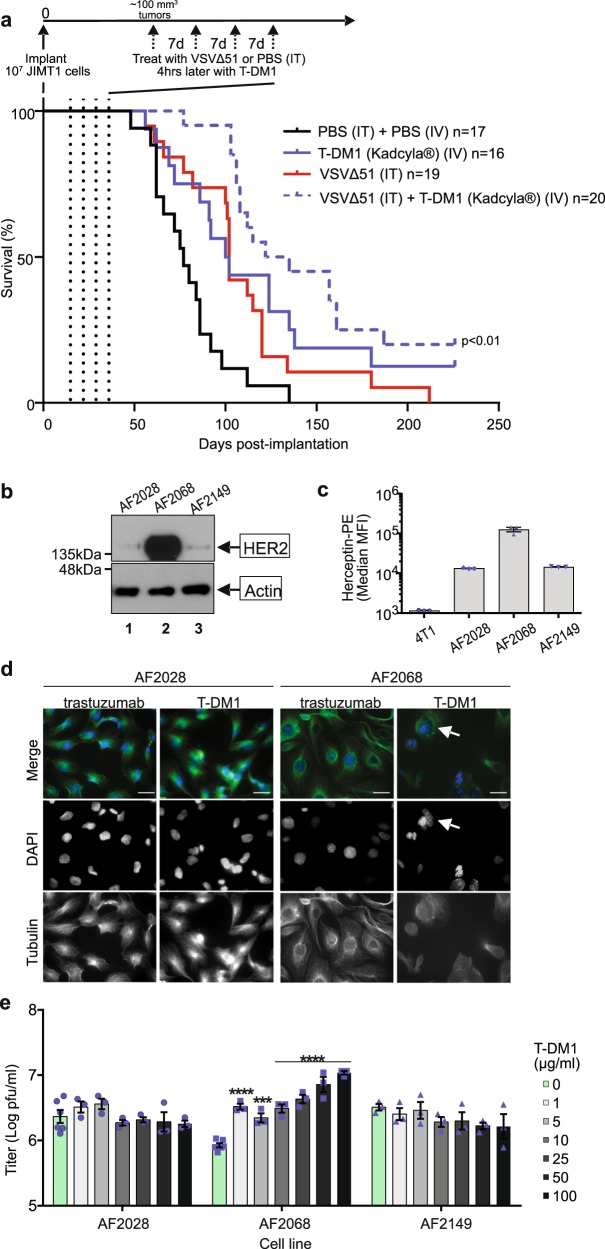
T-DM1 increases oncolytic activity of VSVΔ51 in vivo and in human patient samples. **a** BALB/c nude mice were implanted with 1 × 10^7^ JIMT1 cells. Once tumors were ~100 mm^3^, mice were treated weekly four times (dotted lines) with the regimen of VSVΔ51-Fluc (1E8 pfu) or PBS (intratumorally) followed by 10 mg/kg T-DM1 (Kadcyla®) or PBS 4 h later (intravenously). Survival was monitored over time (*n* = 16–20 mice per group as indicated, pooled over three independent experiments) and was observed to be significantly reduced with the combination of VSVΔ51 + T-DM1 compared to either monotherapies (*p* = 0.039 compared to Kadcyla; *p* = 0.004 compared to VSV; *p* < 0.0001 compared to PBS, Gehan–Breslow–Wilcoxon test). **b** Ascites fluid cells derived from three different human ovarian cancer patients were each lysed and blotted for HER2 or actin as a loading control. **c** Mouse breast carcinoma 4T1 cells, and the ascites fluid-derived cell lines were subject to extracellular staining and flow cytometry. Cells were stained with Herceptin® (trastuzumab, 1:1000), followed by goat anti-human IgG-PE (1:100). PE signal (*y* axis) was analyzed by flow cytometry, median MFI are shown; *n* = 3 ± SD. **d** AF2028 and AF2068 were plated on glass coverslips, treated with 100 µg/ml T-DM1 or trastuzumab, then fixed and stained for β-tubulin and DAPI 24 h later. Objective = ×63, scale bar = 50 µm, *n* = 3. White arrow indicates polynuclear cells. **e** The ascites fluid-derived cell lines were each pretreated with 0–100 µg/ml of T-DM1 for 4 h, washed and infected with VSVΔ51 MOI 0.01 and supernatants tittered by plaque assay after 45 h (*n* = 3 mean ± SEM; one-way ANOVA with Dunnett’s multiple comparisons test was performed and compared to 0 µg/ml condition for each cell line).

It has been previously shown that treatment with microtubule destabilizers in vivo leads to profound vascular shutdown^31^. In fact, a number of microtubule stabilizers are being used as vascular disrupting agents^32^. However, we have also previously shown that vascular disruption can preclude spread and delivery of VSVΔ51 throughout the tumor in vivo^33^. For this reason, in prior studies we have established a treatment protocol in tumor-bearing mice, where VSVΔ51 is administered 4 h before the MDA^12^. We used a similar approach in order to evaluate the combination of T-DM1 with VSVΔ51 in a murine model of HER2^+^ cancer, where JIMT1 trastuzumab-resistant human breast cancer xenografts were implanted subcutaneously into nude mice and allowed to grow to ~100 mm^3^. Tumor-bearing mice were treated with 1E8 pfu VSVΔ51, or PBS, intratumorally to ensure virus delivery, followed by 10 mg/kg Kadcyla® (T-DM1), or PBS, intravenously, 4 h later. This regimen was administered four times, 7 days apart, and survival monitored over time. The combination therapy of T-DM1 with VSVΔ51 significantly improved survival compared to monotherapies (*p* < 0.0001 compared to PBS; *p* = 0.039 compared to Kadcyla alone; *p* = 0.004 compared to VSVΔ51 alone; Gehan–Breslow–Wilcoxon test, Fig. 3a).

### T-DM1 increases VSVΔ51 oncolysis in HER2^+^ patient samples

Given that the combination of MDAs and VSVΔ51 was previously found to be effective in a murine transgenic model of ovarian cancer^12^, we next examined the relevance of our findings to the treatment of human ovarian cancer. We had access to three patient ascites-derived ovarian cancer cell lines. Upon pretreatment of all three cell lines (AF2028, AF2068 and AF2149) with 100 nM colchicine, there was a robust increase in VSVΔ51-GFP output as measured by fluorescence microscopy (Supplementary Fig. 9a) and viral titer (~10–100-fold, Supplementary Fig. 9b). Western blotting for HER2 (Fig. 3b, Supplementary Fig. 6), flow cytometry (Fig. 3c, Supplementary Fig. 2) or immunofluorescence staining using trastuzumab as a primary antibody (Supplementary Fig. 9c) revealed that only AF2068 overexpressed this protein. Treatment of AF2068 with 100 µg/ml T-DM1 resulted in microtubule disruption and evidence of polynucleation as revealed by immunofluorescence staining for tubulin (Fig. 3d). Strikingly, following administration of up to 100 µg/ml of T-DM1, concentrations reflective of recommended clinical dosages, only the HER2-overexpressing AF2068 cells demonstrated a significant ~10-fold boost in VSVΔ51 titer as determined by plaque assay (Fig. 3e) resulting in increased cell death (Supplemental Fig. 9d). Taken together our data demonstrate increased sensitivity of HER2-overexpressing patient-derived cell lines to oncolysis induced by the combination of T-DM1 and VSVΔ51, further supporting the clinical relevance of administering T-DM1 in combination with oncolytic VSV in HER2-overexpressing tumors.

## Discussion

The resistance of tumors to oncolytic virotherapy is well recognized as a hurdle to their clinical success^11,34^. This is why our group and others have spent the last decade devising small molecule-based approaches to sensitize tumors to oncolytic virus infection. This approach, in conjunction with highly attenuated and safe viruses (like VSVΔ51) has the main advantage that it physically separates the oncolytic virus and its enhancing component, giving greater control over infection, ensuring virus safety while promoting anti-cancer efficacy. By the same token, one of the main perceived limitations for this combination approach is toxicity and the potential for off-target effects in normal cells. In an attempt to overcome these limitations, we have harnessed antibody–drug conjugation (ADC) technology as a strategy to enable targeting of these small molecules to HER2^+^ tumors.

Using the clinically approved T-DM1 as proof of concept, we found, that delivering the potent DM1 microtubule destabilizing payload via trastuzumab increases viral spread and bystander killing in HER2^+^ human cancer cells, analogously to what we have previously shown with “free” small molecule MDAs like colchicine (Figs. 1 and 2). Importantly, T-DM1 did not alter virus growth in normal fibroblasts nor did it lead to substantial bystander killing of those cells (Fig. 1e, Supplementary Fig. 4). Furthermore, DM1-mediated microtubule disruption was dependent on HER2 expression as it was not detected in human HER2-negative murine 4T1.2 cells but was observed in human HER2-overexpressing 4T1-2-HER2 cells, which also demonstrated increased VSVΔ51-mediated oncolysis (Supplementary Fig. 8). Finally, we demonstrate that the combination of VSVΔ51 and T-DM1 significantly improves survival in a human xenograft model of trastuzumab-resistant breast cancer (Fig. 3a) and increases VSVΔ51 growth specifically in HER2-overexpressing ovarian cancer cells derived from ascites obtained from patients (Fig. 3b–e, Supplementary Fig. 9). It is to be noted that while most human cells express detectable levels of HER2, we demonstrate here that HER2-overexpressing cancer cells are more sensitive to lower and clinically relevant concentrations of T-DM1, promoting sensitization to oncolytic VSVΔ51. This further suggests the applicability of this combination regimen towards the treatment of HER2^+^ cancers more generally, potentially beyond the approved T-DM1 indication in breast cancer.

The repertoire of clinical-stage ADCs continues to expand. While a handful of ADCs have been approved for the treatment of cancer, hundreds of mAbs and ADCs are currently in preclinical studies evaluating a number of different target antigens specific to cancer cells or expressed within the tumor stroma or vasculature^20,35^. We would expect that conjugation of tubulin inhibitors to other targeted antibodies (e.g. CD30, Adcetris®) would also be effective in increasing the oncolytic efficacy of rhabdoviruses in these tumor types. While effective with oncolytic rhabdoviruses like VSV and Maraba, T-DM1 may not be broadly applicable to all oncolytic viruses given MDAs do not similarly enhance the growth of DNA viruses such as vaccinia and HSV-1^12^. Nevertheless, our study provides proof of concept that linking small molecule VSes to targeted antibodies is a viable strategy to synergize with oncolytic rhabdoviruses such as VSVΔ51.

From our data, it emerges that not only does T-DM1 sensitize HER2^+^ tumors to oncolytic virus infection, but also that oncolytic VSV provides added benefit in the context of trastuzumab-refractory tumors. This may provide new options for patients that have developed trastuzumab resistance but for which HER2 remains expressed at the surface as is observed in JIMT1. Indeed, the fact that T-DM1 is an approved drug for HER2^+^ breast cancer and that both VSV and Maraba are undergoing clinical evaluation for solid tumors including breast cancer (NCT02923466, NCT02285816) provides a clear path to clinical translation of this approach.

In short, we demonstrate here for the first time that combining an ADC like T-DM1 with VSVΔ51 can increase viral infection and cytokine-mediated bystander killing of HER2^+^ tumor cells. Systemic delivery of T-DM1 in combination with oncolytic VSV delayed tumor progression in HER2-overexpressing, but trastuzumab-refractory, JIMT1 human breast cancer xenografts compared to monotherapies, underscoring the potential of this combination in treating both oncolytic virus- and trastuzumab-refractory malignancies. To our knowledge, this is the first proof-of-concept of using an antibody to direct a systemically delivered chemically conjugated viral-sensitizing drug to a tumor, in order to specifically enhance oncolytic virotherapy.

## Methods

### Cell lines

786-0 (human renal carcinoma), GM38 (normal human fibroblasts), T47D (human mammary epithelial), MCF7 (human mammary adenocarcinoma), 4T1 (mouse mammary epithelial) and Vero (African Green Monkey kidney, CCL-81) cells were obtained from the American Type Culture Collection (ATCC) and maintained in Dulbecco’s modified Eagle’s medium (DMEM, HyClone, Waltham, Massachusetts or Corning, Manassas, Virginia) with 10% fetal bovine serum (FBS; VWR, Mississauga, Ontario). SKOV3 (human ovarian) was obtained from ATCC and maintained in Roswell Park Memorial Institute medium (RPMI; ATCC, Cat. # ATCC 30-2001) with 10% FBS. JIMT1 (human herceptin-resistant breast cancer, ACC589) cells were obtained from the Leibniz Institute DSMZ-German Collection of Microorganisms and cultured in DMEM with 10% FBS. Primary human ovarian cancer cells (AF2028, AF2068, AF2149) were obtained from the Ottawa Ovarian Cancer Tissue Bank under a protocol approved by the Ottawa Health Science Network Research Ethics Board (OHSN-REB 1999540-01H) with informed consent. Cells were isolated from ascites fluid collected from patients with ovarian cancer and maintained in primary culture in RPMI medium supplemented with 10% FBS. 4T1.2 and 4T1.2-HER2 were obtained from the laboratory of Dr. Michael Kershaw^30^ and were cultured in RPMI with FBS. All media were supplemented with 1% penicillin–streptomycin solution (ThermoFisher Scientific, Cat. # 15140163). All cells were incubated at 37 °C in a 5% CO_2_ humidified incubator, routinely tested for mycoplasma contamination by Hoechst staining and PCR (Diamed, Mississauga, Ontario, Catalog # ABMG238) and used within 3–10 passages since thaw.

### Viruses, purification and quantification

The oncolytic versions of VSV (Indiana serotype) encoding a firefly luciferase protein tag (VSVΔ51-Fluc) or encoding a green fluorescent protein (VSVΔ51-GFP) tag were grown and titered on Vero cells as previously described^29,36^. Briefly, VSVΔ51 was added at an MOI of 0.01 to 95% confluent Vero cells in 150 mm culture dishes or roller bottles in a total volume of 25 ml complete DMEM. Inoculated Vero cells were incubated at 37 °C with 5% CO_2_ for 24 h or until ~50% CPE (cytopathic effects, cell rounding) was observed. Supernatants were collected and pelleted at 780×*g* to clear heavy debris. Virus contained within the cleared supernatant was subsequently subject to 0.22 µm membrane filtration and purified using 5–50% Optiprep (Sigma-Aldrich, Oakville, ON, Canada, Cat. # D1556) gradient^36^. The purified virus suspension was aliquoted and frozen at −80 °C. For all virus infections, viruses were diluted in serum-free DMEM to obtain the specified MOI, or for mock infection cells were supplemented with an equal volume of serum-free DMEM. For high-throughput luciferase titering^29^, Vero cells were prepared to be 95–100% confluent in opaque white 96-well plates in 100 μl complete DMEM supplemented with 30 mM HEPES. VSVΔ51-Fluc infected samples to be titered were transferred (25 µl/well) onto the Vero cells along with a standard curve prepared from a purified virus stock of known titer and diluted from 10^8^–10^1^ PFU/ml in duplicate for each 96-well plate. Vero plates were then incubated for 5 h at 37 °C 5% CO_2_, following which a d-luciferin (PerkinElmer, Waltham, MA, USA, Cat. # 122799) solution was prepared (2 mg/ml in sterile PBS). Following priming of the Biotek Synergy microplate reader, plates were inserted into the instrument and the d-luciferin solution was automatically dispensed at 25 µl per well. Luminescence was read at an appropriate fixed sensitivity. Standard curve values allow for the generation of a Hill equation which was applied to the titered samples to obtain Viral Expression Units (VEU) using R software. For virus titration using standard plaque assay, Vero cells were seeded into 12-well plates at a final density of 3E5 cells per well. Infectious supernatants were serially diluted using serum-free DMEM, transferred (500 µl per well) onto Vero cells and incubated at 37 °C, 5% CO_2_ for 45 min, following which media was removed and replaced with 1 ml per well of an agarose overlay (1:1 ratio of 1% agarose mixed with 2× DMEM containing 20% FBS). After a 24 h incubation, plaques were fixed with methanol:glacial acetic acid in a 3:1 ratio for a minimum of 1 h, then stained for 30 min with a Coomassie Blue solution (4 g Coomassie Brilliant Blue R (Sigma, cat. B0149), 800 ml methanol, 400 ml acetic acid and 2800 ml distilled water) to visualize and count plaques. For quantification of viral spread, 6-well plates were treated and infected as described, overlayed with an agarose solution, fixed and stained with Coomassie blue after 24–72 h. Plaque diameters were quantified using ImageJ software.

### Drugs, antibodies, cytokines

Trastuzumab (Herceptin®; Hoffman-La Roche, Mississauga, Ontario, Canada), T-DM1 (Kadcyla®; trastuzumab emtansine; Hoffman- La Roche) and IVIG (Gamunex®; 10% immune globulin intravenous (human), Grifols, Mississauga, Ontario, Canada, DIN 02247724) were obtained from clinical preparations at the Ottawa Hospital Pharmacy, stored at 4 °C and used at the indicated concentrations. Colchicine (Sigma-Aldrich, Cat. # C9754) was resuspended in 100% DMSO to 100 mM and was stored at −80 °C and diluted to 100 µM in DMSO before use. Recombinant human TNFα (R&D Systems, Oakville, Ontario, Canada, Cat. # 210-TA) was resuspended in sterile PBS with 0.1% BSA and stored at −20 °C. All compounds were diluted to specified conditions in serum-free media for all assays. For competition assays, 786-0 cells and JIMT1 cells were seeded in 24-well plates and incubated overnight at 37 °C in a 5% CO_2_ humidified incubator. Cells were then pretreated with trastuzumab or IVIG (786-0 at 1000 μg/ml, JIMT1 at 250 μg/ml), or mock-treated for 2 h. Next, supernatants were aspirated and cells were treated with equivalent concentrations of T-DM1 for 2 h. Subsequently, cells were washed once with PBS and infected with VSVΔ51-GFP at MOI 0.01 for 45 h.

### Cell viability assay

The metabolic activity of the cells was assessed using alamarBlue (BioRad, Mississauga, Canada) or resazurin sodium salt (Sigma-Aldrich) according to the manufacturer’s protocol. Treated and/or infected cells were administered 10% (volume/volume, final) resazurin in each well and incubated for 2–4 h, depending on the cell line. Fluorescence was measured at 590 nm upon excitation at 530 nm using the Fluoroskan Ascent FL (Thermo Labsystems, Beverly, MA) or the BioTek Microplate Reader (BioTek, Winooski, VT, USA).

### IFNβ ELISA

786-0 cells were plated to confluency in 12-well plates and incubated overnight 37 °C in a 5% CO_2_ humidified incubator. Cells were then treated with specified drug concentrations and infected 4 h later with VSVΔ51-GFP at an MOI of 0.1 or mock infected. Twenty hours post infection, cell supernatant was collected and frozen with 1:100 protease inhibitor complex (Roche, Cat. # 11697498001). Verikine human IFNβ ELISA (PBL Interferon Source, Piscataway, NJ, USA, Cat. # 41410) kits were used following the manufacturer’s instructions and IFNβ values (pg/ml) were interpolated from the obtained standard curve.

### Cell lysis and western blotting

Whole-cell lysates were obtained by lysing the cells in 50 mM Hepes, pH 7.4, 150 nM NaCl, 2 mM Na_3_VO_4_, 10 mM EDTA, 100 mM NaF, 10 mM Na_2_P_2_O_7_, protease inhibitor cocktail (Roche) and 1% Triton-X 100 on ice. Protein concentration was determined by Bradford assay (BioRad, Cat. # 5000002) and 10–20 µg of cell extract were run using the NuPAGE SDS-PAGE system (Invitrogen, Carlsbad, CA, USA, Cat. # NP0322) and transferred onto a nitrocellulose membrane (BioRad, Cat. # 1620115). Membranes were blocked with 5% milk in TBS-T and probed with a rabbit polyclonal antibody to β-actin (1:5000, Cell Signaling Technology (CST), Danvers, MA, USA, Cat. # 49705) as a loading control, or a mouse monoclonal antibody to human HER2/ErbB2 (1:1000, Invitrogen, Cat. # MA5-13105) followed by incubation with horseradish-peroxidase conjugated rabbit or mouse secondary antibodies (1:5000), respectively (Jackson ImmunoResearch Laboratory, West Grove, PA, USA, Cat. # 711-035-152 (anti-rabbit) 715-035-150 (anti-mouse)). Supersignal West Pico Plus Chemiluminescent substrate (Thermo Scientific, Burlington, Ontario, Canada, Cat. # 34577) was used to visualize the protein bands.

### Immunofluorescence staining

Cells were plated on sterile glass coverslips in 12-well plates and incubated overnight 37 °C in a 5% CO_2_ humidified incubator. Cells were then treated at the specified drug concentrations for 4 h and infected with VSVΔ51-GFP at an MOI of 0.01 or mock infected. Twenty-four hours post infection, cells were fixed with 4% paraformaldehyde, quenched with 100 mM glycine in PBS* (supplemented with 1 mM CaCl_2_ and 0.5 mM MgCl_2_), permeabilized with 0.1% Triton-X 100, then blocked in 5% BSA in PBS*. Slides were subsequently incubated overnight at 4 °C in a humidified chamber with a goat anti-β tubulin primary antibody (1:200, Abcam, Cat. # AB6046) or mouse anti-α-tubulin (1:50, Santa Cruz Biotechnology, Dallas, TX, USA, Cat. # SC-8035), in 1% bovine serum albumin (BSA)-PBS*. Following washes, coverslips were incubated for 1 h at room temperature with a secondary donkey anti-goat antibody conjugated to Alexa 594 (Invitrogen, Cat. # A-11058) or goat anti-mouse antibody conjugated to Alexa 488 (Invitrogen, Cat. # A-11001) diluted 1:200 in 1% BSA. Coverslips were mounted on slides with ProLong Gold Antifade reagent with DAPI (Life technologies/Thermo Scientific, Burlington, Ontario, Cat. # P36930) and stored at 4 °C. Images were taken using the AxioCam HRm camera (Carl Zeiss Ltd, Toronto ON) mounted on the Zeiss Axioscope Imager M1 microscope. Polynuclear and infected cells were manually counted from 6 fields per experimental condition, in triplicate at 40× with a minimum of 100 cells counted per condition.

### Flow cytometry

1 × 10^6^ cells were resuspended in 200 µl of FACS buffer (0.5% BSA-PBS) and transferred to round-bottom 96-well plates and stained with Fixable Viability Dye 510 (BD Horizon, San Jose, California, USA, Cat. # 564406) at 1:1000 for 30 min at 4 °C in the dark. Cells were then pelleted at 1500 rpm, 5 min, at 4 °C, washed with FACS buffer, then stained with trastuzumab at 1:1000 in FACS buffer (final concentration 21 µg/ml) for 60 min at 4 °C in the dark. Cells were then pelleted, washed, and stained with goat anti-human IgG-PE (Invitrogen, Cat. # PA1-86978) at 1:100, for 60 min at 4 °C in the dark. Cells were then washed, pelleted, and resuspended in 1% PFA-PBS and stored overnight at 4 °C. Immediately prior to acquisition, samples were filtered (BioDesign Inc., Carmel, New York, USA, N50R CellMicroSieves, 50 µm pore size) then subjected to flow cytometry using a BD LSRFortessa Flow Cytometer (BD Horizons). Data were analyzed using FlowJo v10.6 software. Unstained controls were prepared in parallel, and PE-treated beads were used for compensation and gating.

### JIMT1 xenograft model

Six- to eight-week-old female CD1 nude mice (Crl:CD1-*Foxn1*^*nu*^; Charles River Laboratories, Wilmington, Massachusetts, USA, Strain code # 086) were implanted subcutaneously with 1E7 JIMT1 cells resuspended in 100 µl PBS and 100 µl GelTrex (ThermoFisher, Cat. # A1413201), for a 200 µl total volume. When tumors reached approximately 100 mm^3^, mice were treated with VSVΔ51-Fluc (1E8 pfu) or PBS i.t. and 4 h later with 10 mg/ml Kadcyla® or PBS i.v. This regimen was repeated four times, a week apart. Tumors were measured every other day using an electronic caliper and volumes were calculated as (length × width^2^)/2. Mice were culled when tumor volumes reached above 1500 mm^3^, and according to the institutional guidelines review board for animal care. In vivo experiments were performed via protocols OHRI-2265 and OHRI-2264, which are in good standing with the Animal Care Committee, and care and treatment of animals was in accordance with the ethical standards of the Canadian Council on Animal Care and with the Animals for Research Act. Each treatment group (PBS/PBS, PBS/T-DM1, VSVΔ51-Fluc/PBS, VSVΔ51-Fluc/T-DM1) comprised *n* = 16–20 nude mice, pooled over three independent experiments. Mice which failed to develop growing tumors by the time of the first treatment were excluded from the studies. Mice-bearing tumors were randomized based on tumor volumes such that the initial average tumor volume at time of treatment was evenly distributed between groups. Studies were not formally blinded, but mouse tumor measurements, assessments and injections were carried out by an animal technician who was not aware of the treatment groups.

### Microarray and analysis

786-0 cells were plated at a density of 1E6 cells in 6-well flat bottom plates. Following overnight growth, cells were pretreated for 4 h with 400 nM Colchicine, 100 µg/ml T-DM1, 100 µg/ml trastuzumab or mock-treated. Following pretreatment, PBS or VSVΔ51-GFP at an MOI of 0.01 was added to cell cultures. Twenty-four hours post infection, RNA was collected using an RNA-easy kit (Qiagen, Valencia, California, USA, Cat. # 74104). Biological triplicates were subsequently pooled and RNA quality was measured using Agilent 2100 Bioanalyzer (Agilent Technologies, Santa Clara, California, USA) before hybridization to Affimetrix Human PrimeView Array (The Centre for Applied Genomics, The Hospital for Sick Children, Toronto, Ontario, Canada). Microarray data were processed using Agilent 2100 Bioanalyzer under default parameters of Gene Level Differential Expression Analysis. A heatmap of expression values and hierarchical clustering was generated using R package pheatmap.

### Quantitative real-time PCR

786-0 cells were pretreated and infected as indicated. Sixteen hours later, cells were collected and RNA extraction was performed by using QiaShredder columns and RNeasy kits (Qiagen). RNA (1 μg) was converted to cDNA using RevertAid First Strand cDNA Synthesis Kit (ThermoFisher Scientific). Real-time PCRs were performed according to the manufacturer’s protocol with the Applied Biosystems PowerUp SYBR Green Master Mix (ThermoFisher Scientific) on a 7500 Fast Real-Time PCR system (Applied Biosystems). Optimal thresholds and reaction efficiencies were established using the Applied Biosystems software and melt curves for each primer exhibited a single peak, indicating specific amplification. *C*_t_ values were determined with the Applied Biosystems software at the optimal threshold for each gene. Gene expression relative to GAPDH was calculated with the Pfaffl method. Fold induction was determined relative to the Mock-treated control for each gene^37^. Primers sequences are as follows:

hIFITM1 F: CCGTGAAGTCTAGGGACAGG

hIFITM1 R: GGTAGACTATCACAGAGCCG

hGAPDH F: ACAGTCAGCCGCATCTTCTT

hGAPDH R: GTTAAAAGCAGCCCTGGTGA

### Statistics and reproducibility

All graphs and statistical analyses were performed using Excel or GraphPad Prism v.6. Means of two groups were compared using two-tailed unpaired Student’s *t*-test. Means of more than two groups were compared by one-way ANOVA with Dunnett’s or Tukey’s multiple correction test. Two-way ANOVA with Sidak or Dunnett’s multiple correction test was applied when groups were split on two independent variables. Normal distribution of the data was assessed using D’Agostino & Pearson omnibus and Shapiro–Wilk normality tests. Alpha levels for all tests were 0.05 (confidence levels of 95%). Biological replicates are indicated by a number *n*, and defined as per NIH guidelines, and error calculated as the standard error of the mean (SEM). Measurements were taken from distinct samples. For all analyses, *p < 0.05, **p < 0.01, ***p < 0.001, ****p < 0.0001; n.s. = not significant. Data were reproduced by at least two different operators.

### Reporting summary

Further information on research design is available in the Nature Research Reporting Summary linked to this article.

## Supplementary information

Supplementary Information

Description of Additional Supplementary Files

Supplementary Data 1

Reporting Summary

## Data Availability

Source data for Figs. 1b–c, e–k, 2b–d, f–g and 3a, c, e, Supplementary Figs. 2c, 3, 4b, 5b, 7a, 8a, d, and 9b, d are in the Supplementary Data 1. Raw and processed microarray data have been deposited in NCBI-Gene Expression Omnibus database (GSE135443). All other relevant data are available from the authors upon request to the corresponding author.
