# Supplementary material for: The strategic combination of trastuzumab emtansine with oncolytic rhabdoviruses leads to therapeutic synergy
Source: Commun Biol. 2020 May 22;3:254. doi: 10.1038/s42003-020-0972-7 (PMC7244474; doi:10.1038/s42003-020-0972-7)
Supplement: Supplementary file 1 — Supplementary Information [file 42003_2020_972_MOESM1_ESM.pdf]

SUPPLEMENTARY INFORMATION

Supplementary Figures

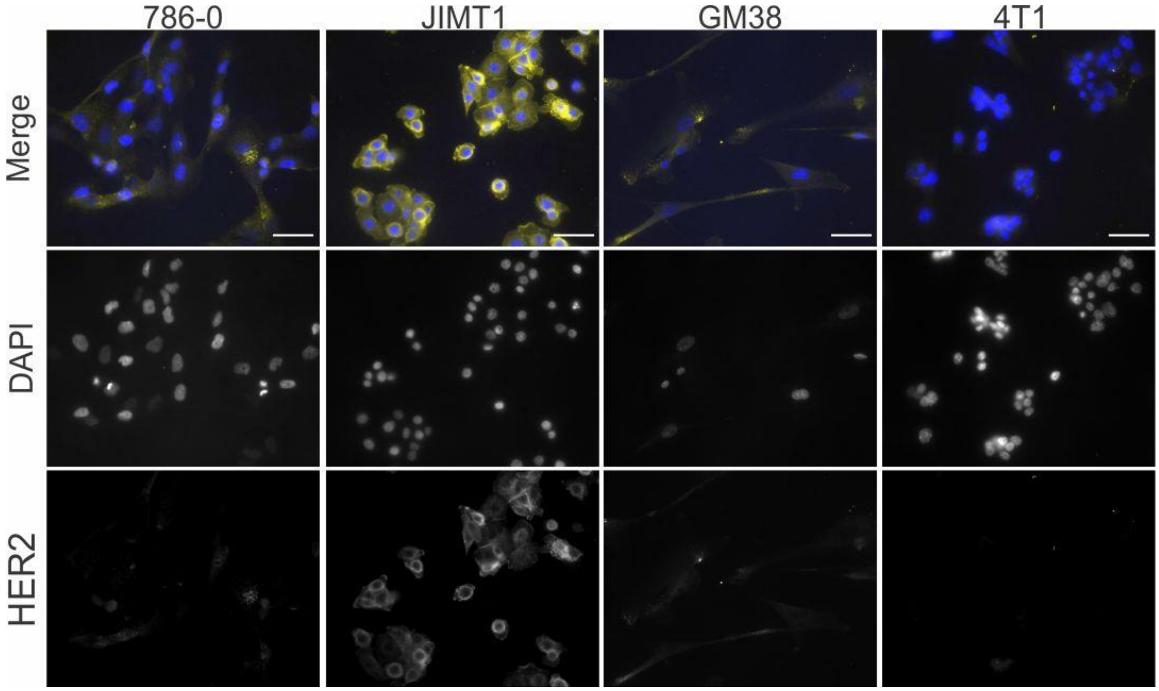

Supplementary Figure 1. Cell surface huHER2 staining in selected cell lines. 786-0 human renal carcinoma cells, JIMT1 human breast carcinoma cells, GM38 normal human lung epithelial cells or murine breast 4T1 cancer cells were seeded on sterile glass coverslips in 12-well dishes. Cells were fixed with 4 % paraformaldehyde, quenched with 100 mM glycine in PBS* (supplemented with 1 mM $CaCl_2$ and 0.5 mM $MgCl_2$), then blocked in 3 % bovine serum albumin (BSA) and 5 % normal goat serum (NGS) in PBS*. Slides were subsequently incubated overnight at 4 °C in a humidified chamber with trastuzumab (1:2000 dilution, 10.5 µg/ml final concentration) (Genentech) in 3% BSA, 5% NGS-PBS*. Following washes, coverslips were incubated for 1 h at room temperature with a secondary goat anti-human antibody conjugated to Alexa 594 (Invitrogen, Cat. # A-11014) diluted 1:400 in 1% BSA. Coverslips were mounted on slides with ProLong Gold Antifade reagent with DAPI (Life technologies/Thermo Scientific, Burlington, ON, Cat. # P36930) and stored at 4 °C. Images were taken using the AxioCam HRm camera (Carl Zeiss Ltd, Toronto ON) mounted on the Zeiss Axioscope Imager M1 microscope. Objective = 40x, scale bar = 50µm, n = 3. Representative images of 3 coverslips per cell line are shown.

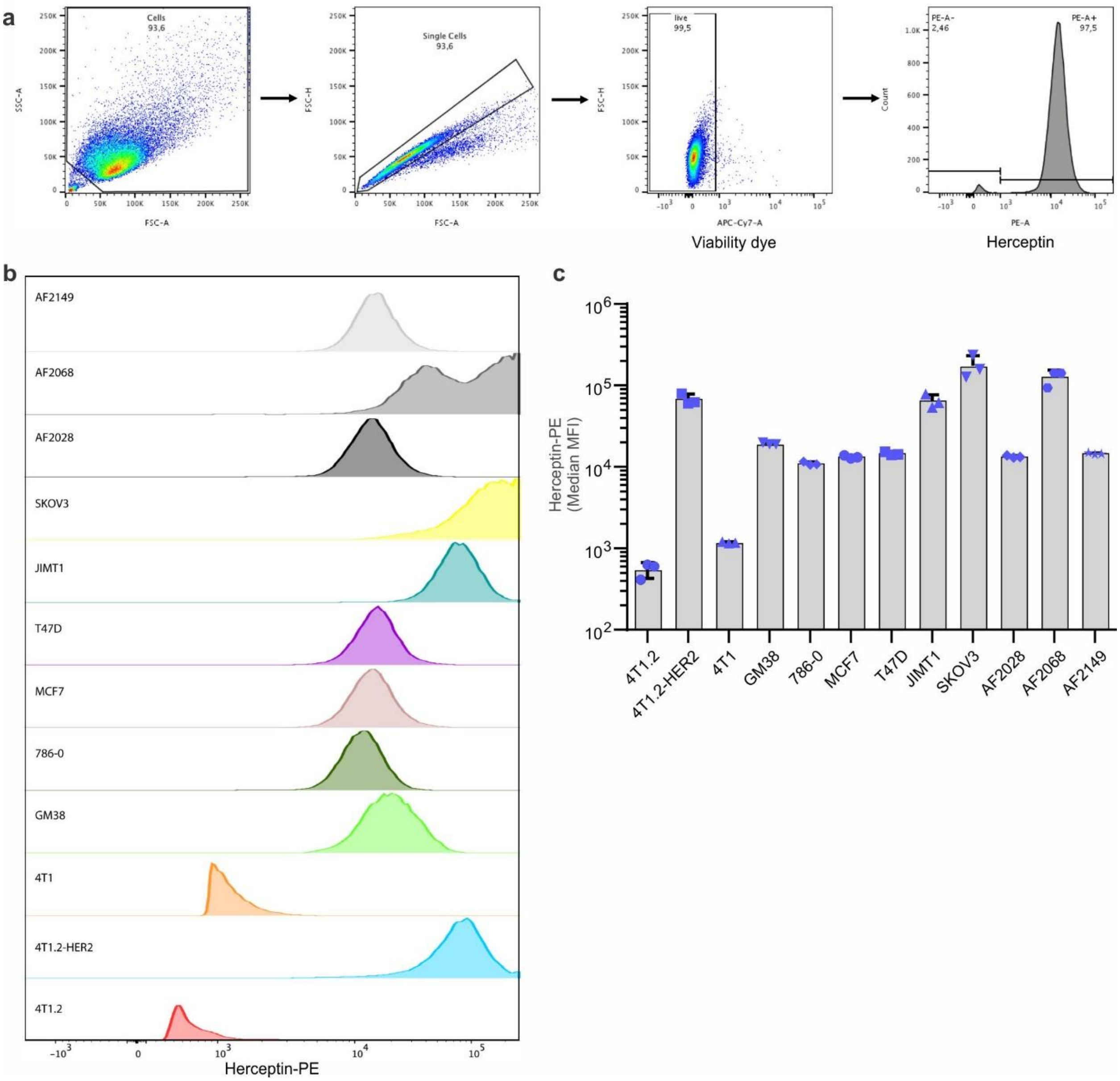

Supplementary Figure 2. Gating strategy and flow cytometry histograms for our panel of cell lines. **a)** Gating strategy for trastuzumab quantification by flow cytometry in our panel of cell lines where 1 x 10^6 cells were resuspended in 200 µl of FACS buffer (0.5 % BSA-PBS) and transferred to round-bottom 96-well plates and stained with Fixable Viability Dye 510 (BD Horizon, San Jose, California, USA, Cat. # 564406) at 1:1000 for 30 minutes at 4 ^{0}C in the dark. Cells were then pelleted at 1500 rpm, 5 minutes, at 4 ^{0}C, washed with FACS buffer, then stained with trastuzumab at 1:1000 in FACS buffer (final concentration 21 µg/ml) for 60 minutes at 4 ^{0}C in the dark. Cells

were then pelleted, washed, and stained with goat anti-human IgG-PE (Invitrogen, Cat. # PA1-86978) at 1:100, for 60 minutes at 4 ^{0}C in the dark. Cells were then washed, pelleted, and resuspended in 1 % PFA-PBS and stored overnight at 4 ^{0}C. Immediately prior to acquisition, samples were filtered (BioDesign Inc., Carmel, New York, USA, N50R CellMicroSieves, 50 μm pore size) then subjected to flow cytometry using a BD LSRFortessa Flow Cytometer (BD Horizons).. Unstained controls were prepared in parallel, and PE-treated beads were used for compensation and gating. **b-c)** Compiled histograms (b) and median MFI (c) for HER2 expression the panel of cell lines presented in this manuscript.

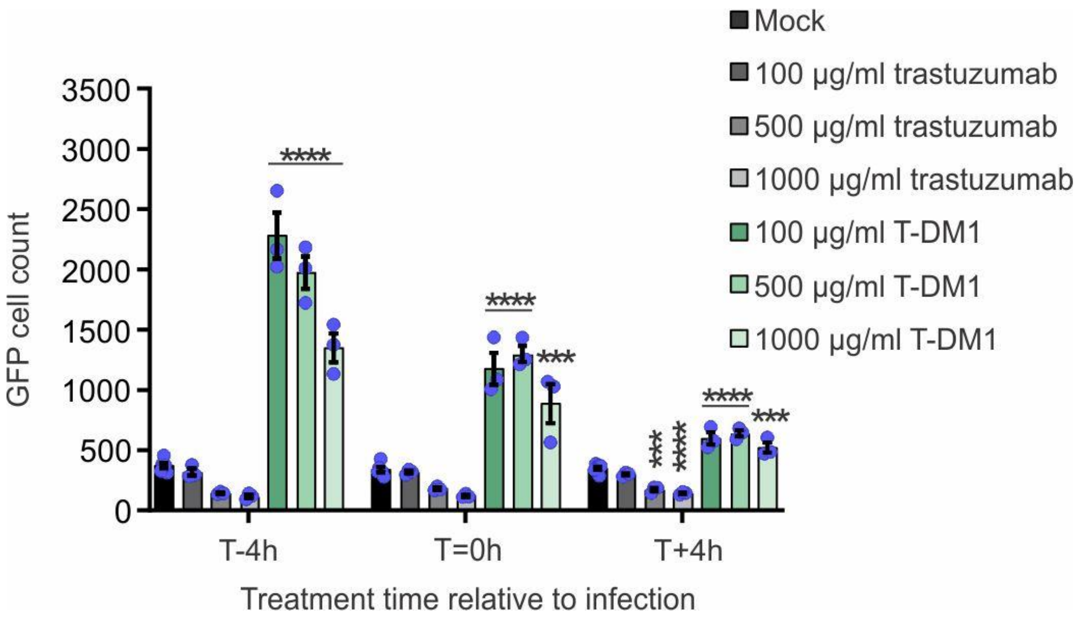

Supplementary Figure 3. T-DM1, but not trastuzumab, increases VSVΔ51-GFP growth in 786-0 cells. 786-0 cells were treated with the indicated amounts of trastuzumab or T-DM1 at 4 h prior to infection (T - 4 h), at the same time as infection (T = 0 h) or 4 h post infection (T + 4 h) with VSVΔ51-GFP MOI 0.01. 24 h later GFP counts were captured by fluorescence microscopy (ArrayScan, ThermoFisher Scientific) and GFP foci quantified (n = 3; mean ± SEM; one-way ANOVA compared to the mock condition was performed for each time point, ***P<0.001, ****P<0.0001).

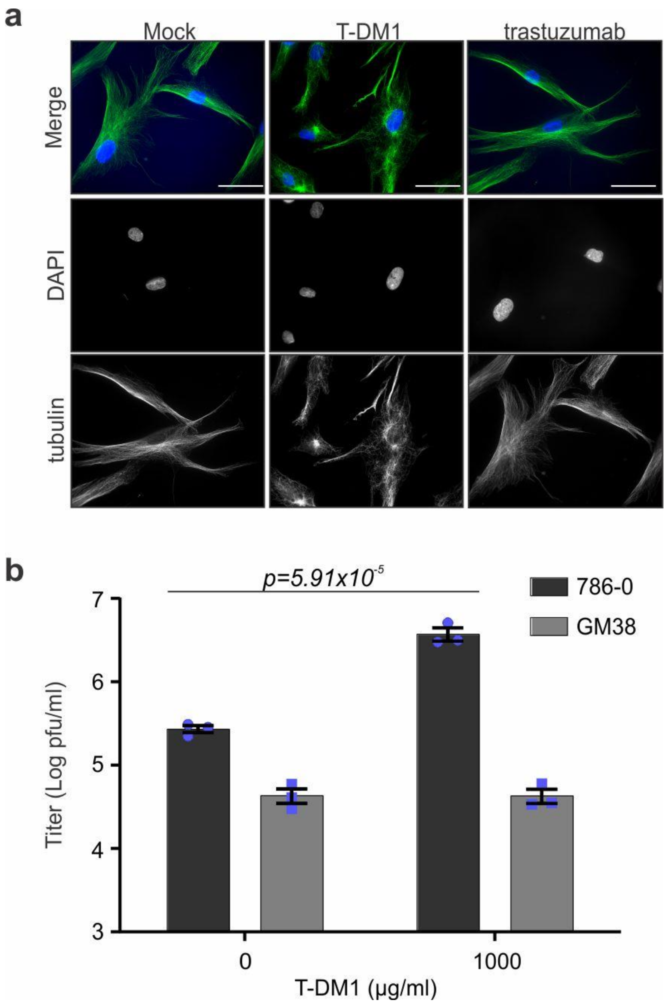

Supplementary Figure 4. T-DM1 does not increase VSVΔ51 viral titers in normal GM38 cells. **a)** GM38 normal human fibroblasts were seeded on sterile glass coverslips in 12-well dishes. Cells were fixed with 4 % paraformaldehyde, quenched with 100 mM glycine in PBS, permeabilized with 0.1 % Triton-X 100, then blocked in 5 % BSA in PBS* (supplemented with 1 mM $CaCl_2$ and 0.5 mM $MgCl_2$). Slides were subsequently incubated overnight at 4 °C in a humidified chamber with goat anti-β-tubulin antibody (abcam, Cat.# AB6046) diluted 1:400 in 1 % bovine serum albumin (BSA)-PBS*. Following washes, coverslips were incubated for 1 h at room temperature with a secondary donkey anti-goat antibody conjugated to Alexa 594 (Invitrogen, Cat. # A-11058) diluted 1:200 in 1 % BSA. Coverslips were mounted on slides with ProLong Gold Antifade reagent with DAPI (Life technologies/Thermo Scientific, Burlington, ON, Cat. # P36930) and stored at 4 °C. Images were taken using the AxioCam HRm camera (Carl Zeiss Ltd, Toronto ON) mounted on the Zeiss Axioscope Imager M1 microscope. Objective = 63X, scale bar = 50 µm, n

= 3. Representative images from 3 coverslips are shown. **b)** Supernatants of 786-0 and normal human GM38 cells pre-treated with 0 or 1000 µg/mL of T-DM1 and infected with VSV51-GFP MOI 0.1 were collected and titered by standard plaque assay. Data represent the average value ± SEM from n = 3 biological replicates, student's t-test was performed for each cell line.

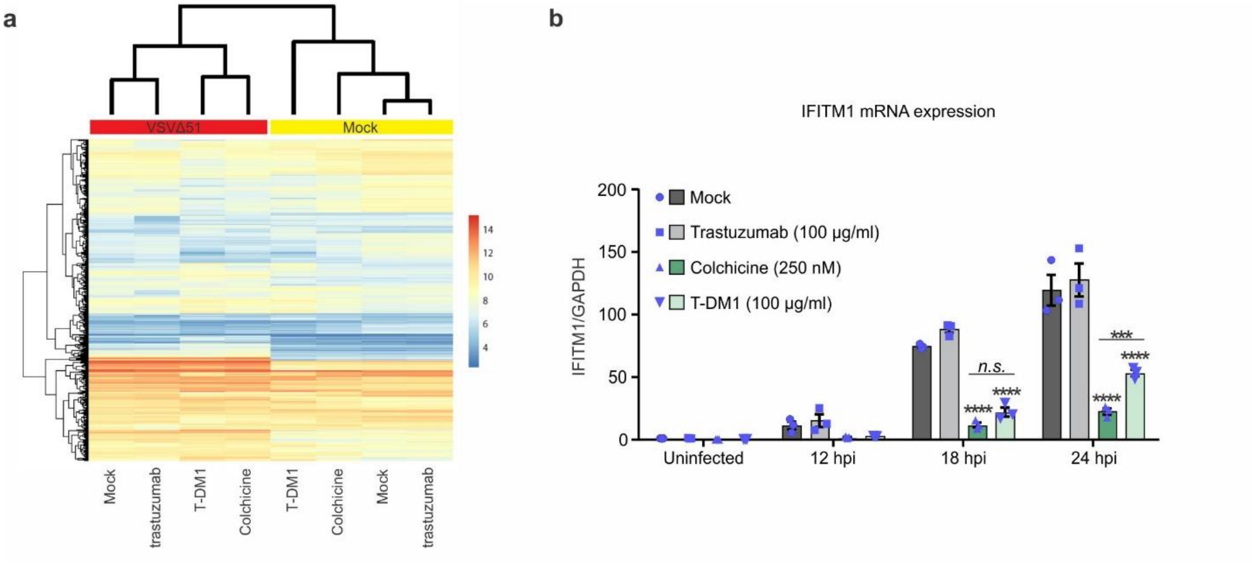

Supplementary Figure 5. Microtubule destabilization induced by T-DM1 or colchicine leads to the suppression of IFN-simulated genes induced by VSVΔ51. **a)** 786-0 were mock-treated or pretreated with either 100 µg/ml trastuzumab, 100 µg/ml T-DM1 or 100 nM colchicine for 4 h, followed by mock infection or infection with VSVΔ51-GFP at MOI 0.1 and RNA collected 24 h later and hybridized on Affymetrix Human PrimeView Array, n = 3 pooled for each condition. Heatmap diagram showing the expression levels of the differentially expressed genes and the hierarchical clustering of the various treatment conditions is presented. **b)** 786-0 were pretreated as indicated for 4 h, after which time cells were washed and infected with VSVΔ51-GFP at MOI 0.01 or mock infected. 12, 18 or 22 h later, RNA was collected, converted to cDNA and qRT-PCR was performed. Data represent the fold change in IFITM1, normalized to GAPDH, relative to uninfected controls (n = 3; mean ± SEM; two-way ANOVA with Tukey's multiple comparisons test was performed, colchicine and T-DM1 conditions were significantly lower than mock or trastuzumab alone at 18 and 24 h post infection (**** $P < 0.001$).

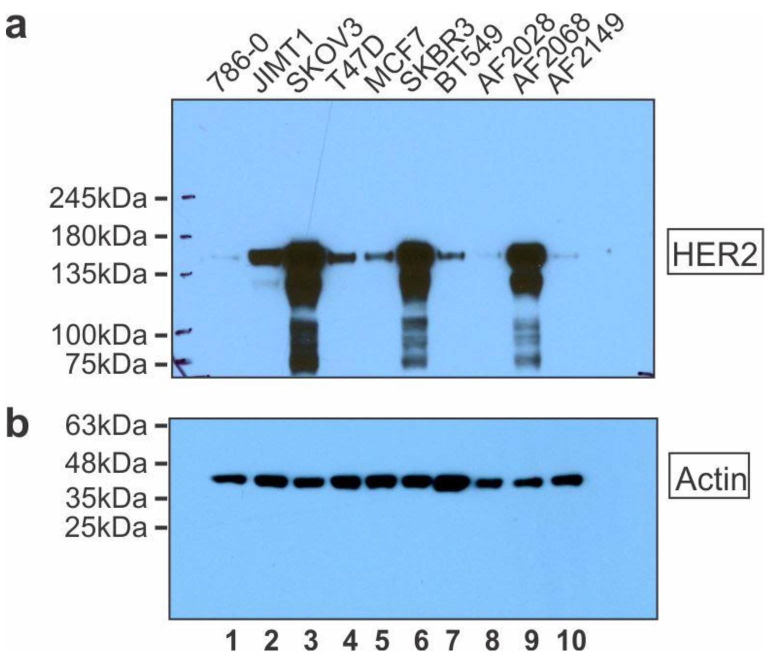

Supplementary Figure 6. Uncropped Western blots for Figures 2a and 3b. A panel of human breast and ovarian carcinoma lines were lysed and subject to Western blotting for human HER2 **(a)** or actin as a loading control **(b)**.

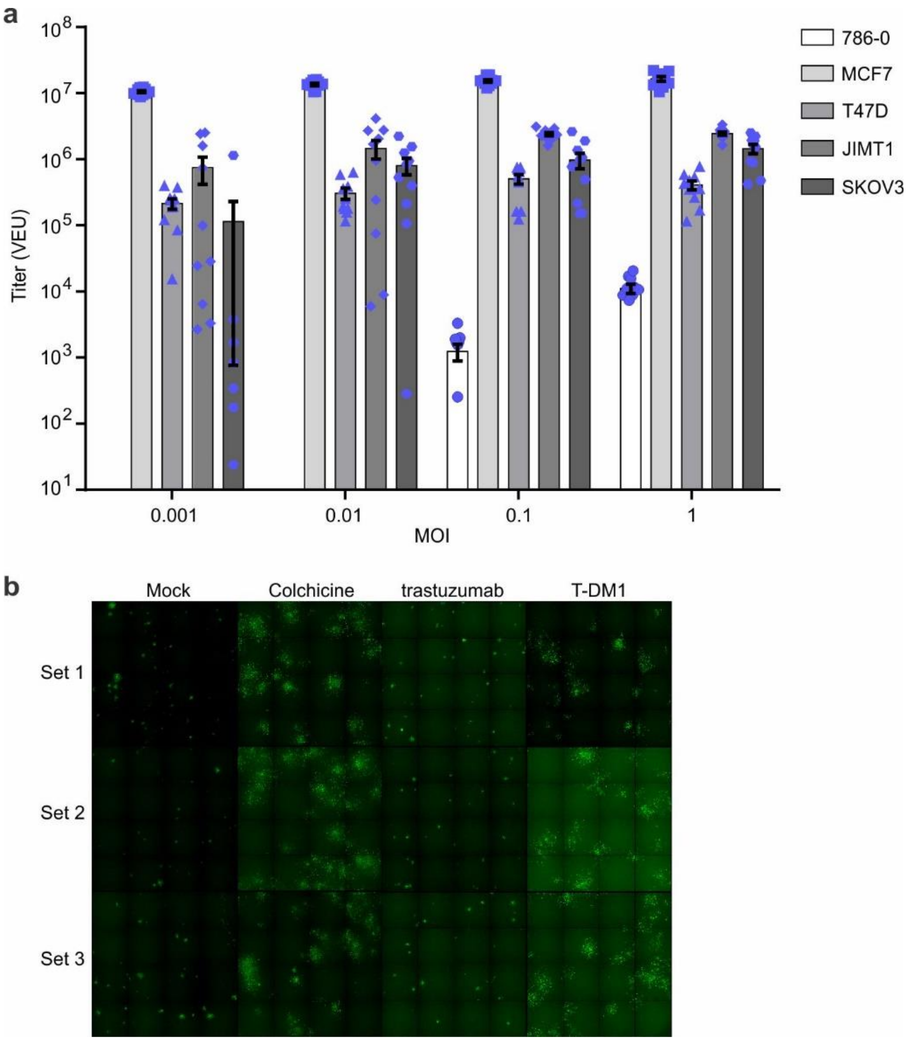

Supplementary Figure 7. Susceptibility of a panel of human cancer cell lines to VSVΔ51 infection. **a)** 786-0, MCF7, T47D, JIMT1 and SKOV3 cells were seeded in 96-well dishes and infected the following day with VSVΔ51-Fluc at MOI 0.001, 0.01, 0.1 or 1. 40 h post-infection supernatants were transferred onto Vero cells and titered by high throughput assay[1] (n = 10; mean ± SEM). **b)** VSVΔ51 spread was evaluated in SKOV3 cells plated in 96-well dishes and pretreated with either 100 nM colchicine, 100 µg/ml T-DM1 or trastuzumab, or mock treated, for 3 h, then washed and infected with VSVΔ51-GFP MOI 0.001 as in Fig. 2d. Media was removed and wells were overlayed with agarose 1 h post-infection, and imaged by Arrayscan high content fluorescent screening microscope (ThermoFisher Scientific, MA, U.S.A.).

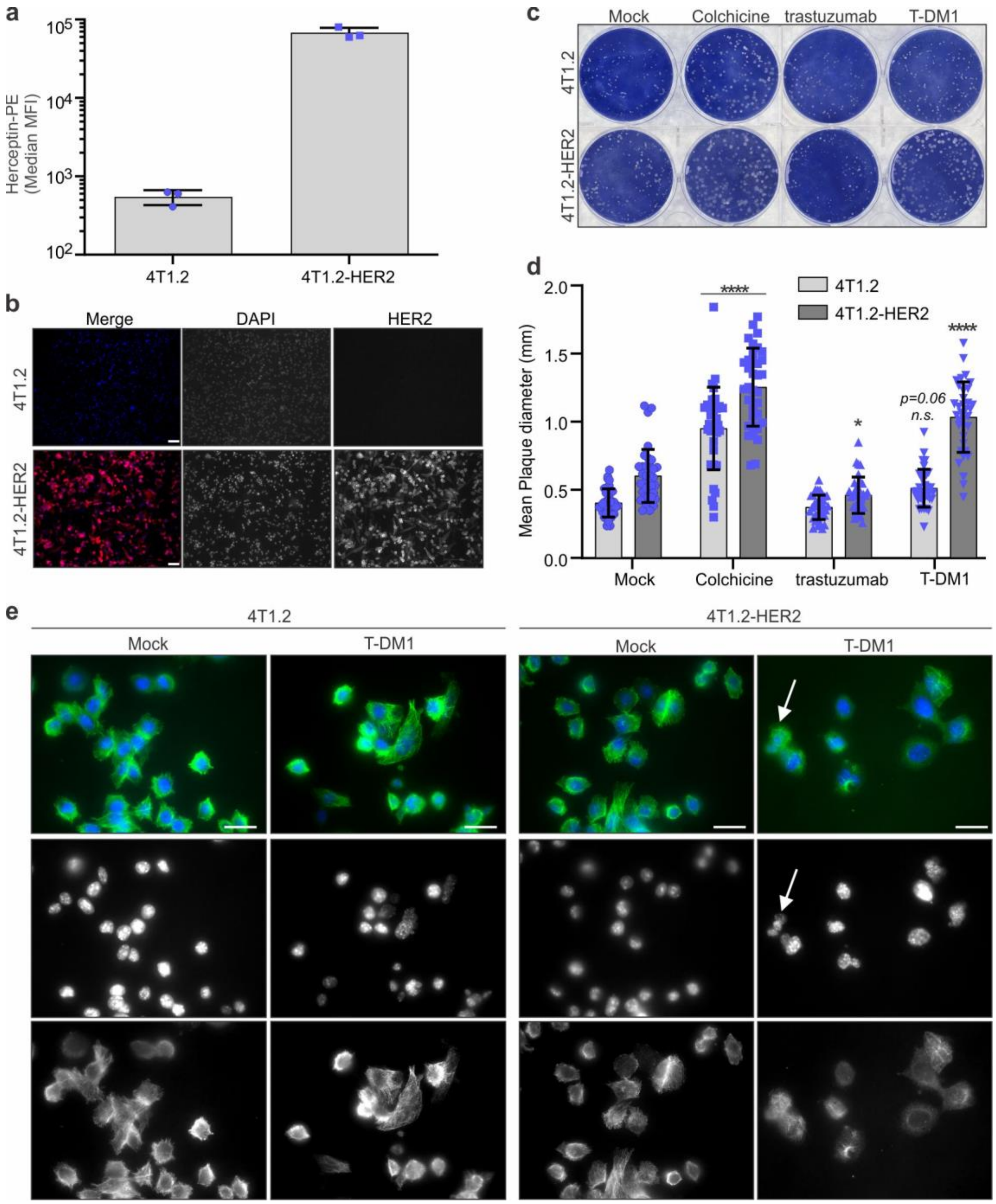

Supplementary Figure 8. T-DM1 increases microtubule disruption and VSVΔ51 viral spread in human HER2-expressing, murine 4T1.2-HER2 cells, with no impact on parental 4T1.2 cells. **a)** 4T1.2 and 4T1.2-HER2 were subject to extracellular staining and flow cytometry. Cells were stained with fixable viability dye (APC-Cy7), followed by staining with Herceptin® (trastuzumab, 1:1000) and goat anti-human IgG-PE (1:300). PE signal (y-axis) was analyzed by flow cytometry,

median MFI are shown from one of three independent experiments ± SD. **b)** 4T1.2 and 4T1.2-HER2 cells were seeded on sterile glass coverslips in 12-well dishes. Cells were stained as in Supplementary Figure 1. Images were taken using the AxioCam HRm camera (Carl Zeiss Ltd, Toronto ON) mounted on the Zeiss Axioscope Imager M1 microscope. Objective = 20X, scale bar = 50µm, n = 3. Representative images of 3 coverslips per cell line are shown. **c-d)** Murine 4T1.2 or 4T1.2-HER2 breast carcinoma cells were seeded in 6-well plates and mock-treated or pretreated with 100 µg/ml T-DM1, 100 µg/ml trastuzumab or 100 nM colchicine for 4 h, washed, and infected with VSVΔ51 MOI 0.001 and overlayed with agarose. Plaques were fixed and stained with Coomassie blue after 48 h. Diameter of 30 plaques per well from n = 3 were measured using ImageJ software and graphed (mean ± SEM; one-way ANOVA with Dunnett's multiple comparisons test compared to Mock for each cell line, ****$P<0.0001$). **e)** Murine 4T1.2 and 4T1.2-HER2 cells were seeded on glass coverslips as in (b), treated with or without 100 µg/ml T-DM1, and stained for β-tubulin as described in Supplementary Figure 4. Images were taken using the AxioCam HRm camera (Carl Zeiss Ltd, Toronto ON) mounted on the Zeiss Axioscope Imager M1 microscope. Objective = 63X, scale bar = 50µm, n = 3. Representative images of 3 coverslips per cell line are shown.

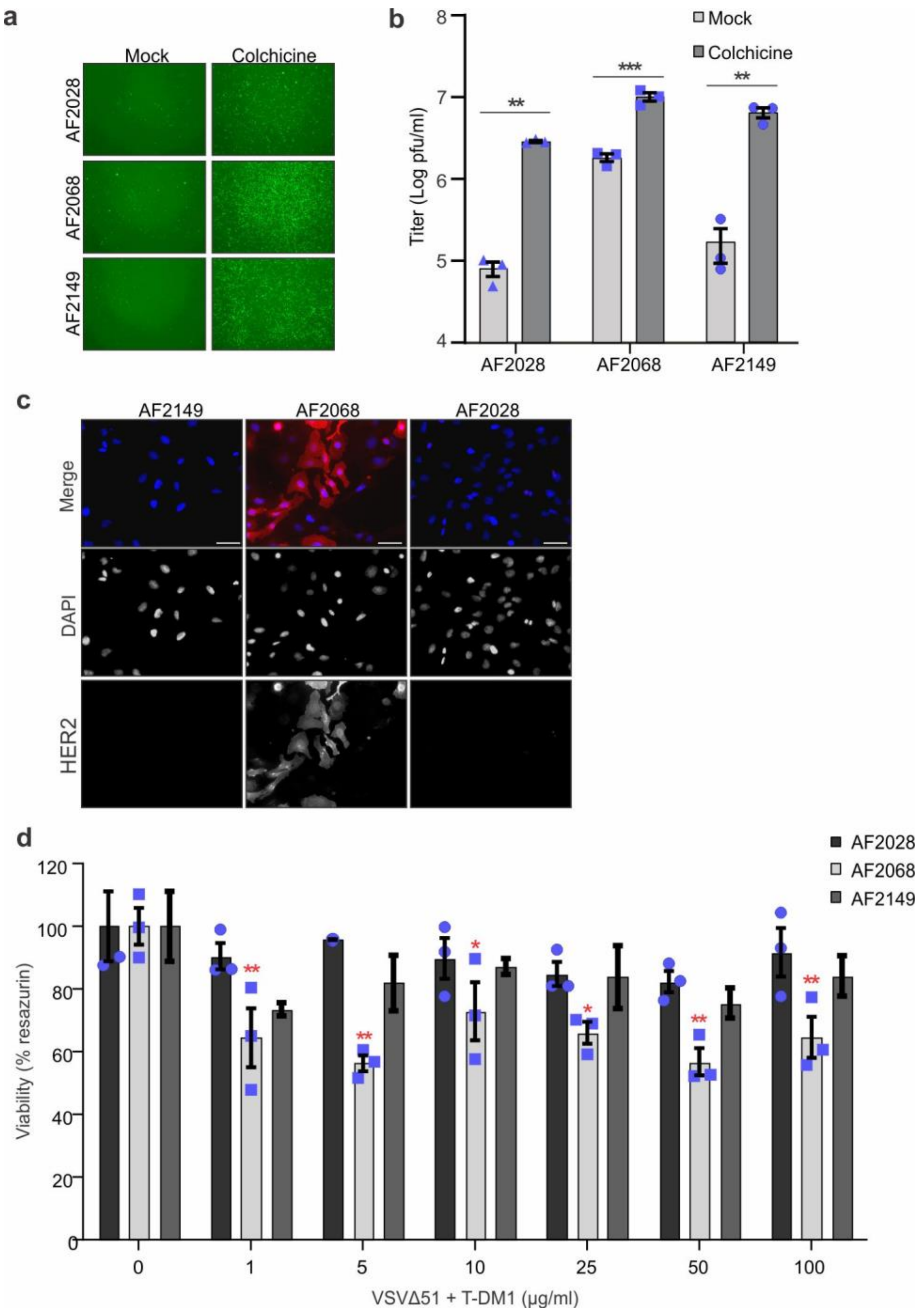

Supplementary Figure 9. Colchicine increases VSVΔ51 viral titers in primary human ovarian cancer cells, but viral oncolysis is augmented only upon HER2-overexpression. **a)** Cells isolated from ovarian cancer patient ascites fluid were plated in 12-well dishes and pretreated with 100 nM colchicine for 4 h prior to infection with VSVΔ51-GFP at MOI 0.01. 45 h later, cells were imaged using fluorescence microscopy at 2X using the EVOS Cell imaging system. A representative image is shown of n = 3 per condition. **b)** Infectious supernatant from (a) was collected and tittered

by standard plaque assay. Data represent the average mean from n = 3 ± SEM, AF2028 **p=0.00336, AF2068 ***p=0.00055, AF2149 **p=0.0067, student's t-test, two-tailed distribution, unequal variance. **c)** AF2028, AF2068 and AF2149 cells were seeded on sterile glass coverslips in 12-well dishes. Cells were stained for β-tubulin as described in Supplementary Figure 4. Images were taken using the AxioCam HRm camera (Carl Zeiss Ltd, Toronto ON) mounted on the Zeiss Axioscope Imager M1 microscope. Objective = 40X, scale bar = 50μm, n = 3. Representative images of 3 coverslips per cell line are shown. **d)** AF2028, AF2068 and AF2149 were pretreated with 0-100 μg/ml T-DM1 for 4 hours, washed and infected with VSVΔ51 MOI 0.01 as in Figure 3d. 45 h post infection, a viability assay was performed and fluorescence measured and normalized to untreated, uninfected controls for each cell line; n=3 mean ± SEM; one-way ANOVA with Dunnett's multiple comparisons test was performed compared to untreated, uninfected controls for each cell line, *P<0.05, **P<0.01).

SUPPLEMENTARY REFERENCES

1 Garcia, V. *et al*. High-throughput titration of luciferase-expressing recombinant viruses. *J Vis Exp*, 51890, doi:10.3791/51890 (2014).
