# Supplementary material for: The strategic combination of trastuzumab emtansine with oncolytic rhabdoviruses leads to therapeutic synergy
Source: Commun Biol. 2020 May 22;3:254. doi: 10.1038/s42003-020-0972-7 (PMC7244474; doi:10.1038/s42003-020-0972-7)
Supplement: Supplementary file 2 — Description of Additional Supplementary Files [file 42003_2020_972_MOESM2_ESM.pdf]

Description of additional supplementary files

File Name: Supplementary Data 1:

Description: Source data for Figure 1b-c, e-k, Figure 2b-d, f-g, Figure 3a, c, e, Supplementary Figures 2c, 3, 4b, 5b, 7a, 8a,d, and 9b, d.
