# Supplementary material for: The strategic combination of trastuzumab emtansine with oncolytic rhabdoviruses leads to therapeutic synergy
Source: Commun Biol. 2020 May 22;3:254. doi: 10.1038/s42003-020-0972-7 (PMC7244474; doi:10.1038/s42003-020-0972-7)
Supplement: Supplementary file 4 — Reporting Summary [file 42003_2020_972_MOESM4_ESM.pdf]

nature research

Corresponding author(s): Jean-Simon Diallo

Last updated by author(s): Apr 17, 2020

Reporting Summary

Nature Research wishes to improve the reproducibility of the work that we publish. This form provides structure for consistency and transparency in reporting. For further information on Nature Research policies, see Authors & Referees and the Editorial Policy Checklist .

Statistics

For all statistical analyses, confirm that the following items are present in the figure legend, table legend, main text, or Methods section.

n/a	Confirmed	
☐	☒	The exact sample size (*n*) for each experimental group/condition, given as a discrete number and unit of measurement
☐	☒	A statement on whether measurements were taken from distinct samples or whether the same sample was measured repeatedly
☐	☒	The statistical test(s) used AND whether they are one- or two-sided<br>*Only common tests should be described solely by name; describe more complex techniques in the Methods section.*
☒	☐	A description of all covariates tested
☐	☒	A description of any assumptions or corrections, such as tests of normality and adjustment for multiple comparisons
☐	☒	A full description of the statistical parameters including central tendency (e.g. means) or other basic estimates (e.g. regression coefficient) AND variation (e.g. standard deviation) or associated estimates of uncertainty (e.g. confidence intervals)
☐	☒	For null hypothesis testing, the test statistic (e.g. *F*, *t*, *r*) with confidence intervals, effect sizes, degrees of freedom and *P* value noted<br>*Give P values as exact values whenever suitable.*
☒	☐	For Bayesian analysis, information on the choice of priors and Markov chain Monte Carlo settings
☒	☐	For hierarchical and complex designs, identification of the appropriate level for tests and full reporting of outcomes
☒	☐	Estimates of effect sizes (e.g. Cohen's *d*, Pearson's *r*), indicating how they were calculated

Our web collection on statistics for biologists contains articles on many of the points above.

Software and code

Policy information about availability of computer code

Data collection	No software was used for data collection.
Data analysis	Agilent 2100 Bioanalyzer and R package pheatmap were used to analyze microarray data.

For manuscripts utilizing custom algorithms or software that are central to the research but not yet described in published literature, software must be made available to editors/reviewers. We strongly encourage code deposition in a community repository (e.g. GitHub). See the Nature Research guidelines for submitting code & software for further information.

Data

Policy information about availability of data

All manuscripts must include a data availability statement. This statement should provide the following information, where applicable:

- Accession codes, unique identifiers, or web links for publicly available datasets
- A list of figures that have associated raw data
- A description of any restrictions on data availability

Source data for Figure 1b-c, e-k, Figure 2b-d, f-g, Figure 3a, c, e, Supplementary Figures 2c, 3, 4b, 5b, 7a, 8a,d, and 9b, d are in the Supplementary Data file. Raw and processed microarray data have been deposited in NCBI-Gene Expression Omnibus database (GSE135443). All other relevant data are available from the authors upon request to the corresponding author.

Field-specific reporting

Please select the one below that is the best fit for your research. If you are not sure, read the appropriate sections before making your selection.

Life sciences study design

All studies must disclose on these points even when the disclosure is negative.

Sample size	All data was generated from at least n = 3 biological replicates. For in vivo studies, no power calculations were performed however numbers of mice used are in keeping with other published studies and are sufficient to detect biologically meaningful effect sizes.
Data exclusions	Animal experiments in some instances excluded animals that failed to develop growing tumours by the time of the first treatment.
Replication	All data was generated from at least n = 3 biological replicates. Data was reproduced by at least two different operators. Microarray data was generated from pooled biological triplicates and subsequently validated by qRT-PCR.
Randomization	Mice bearing tumours were randomized based on tumour volumes such that the initial average tumour volume at time of treatment was evenly distributed between groups.
Blinding	Studies were not formally blinded but mouse tumour measurements and assessments as well as injections were carried out by our animal technician who was not aware of the treatment groups.

Reporting for specific materials, systems and methods

We require information from authors about some types of materials, experimental systems and methods used in many studies. Here, indicate whether each material, system or method listed is relevant to your study. If you are not sure if a list item applies to your research, read the appropriate section before selecting a response.

Materials & experimental systems

n/a	Involved in the study	
☐	☒	Antibodies
☐	☒	Eukaryotic cell lines
☒	☐	Palaeontology
☐	☒	Animals and other organisms
☒	☐	Human research participants
☒	☐	Clinical data

Methods

n/a	Involved in the study	
☒	☐	ChIP-seq
☐	☒	Flow cytometry
☒	☐	MRI-based neuroimaging

Antibodies

Antibodies used	Trastuzumab (Herceptin®; Hoffman-La Roche, Mississauga, Ontario, Canada), T-DM1 (Kadcyla®; trastuzumab emtansine; Hoffman- La Roche) and IVIG (Gamunex®; 10% immune globulin intravenous (human), Grifols, Mississauga, Ontario, Canada, DIN 02247724) were obtained from clinical preparations at the Ottawa Hospital Pharmacy; rabbit polyclonal antibody to beta-actin (1:5000, Cell Signaling Technology (CST), Danvers, MA, USA, Cat. # 49705); mouse monoclonal antibody to human HER-2/ErbB2 (1:1000, Invitrogen, Cat. # MA5-13105); horseradish-peroxidase conjugated rabbit or mouse secondary antibodies (Jackson ImmunoResearch Laboratory, West Grove, PA, USA, Cat. # 711-035-152 (anti-rabbit) 715-035-150 (anti-mouse)); goat anti-beta-tubulin (Abcam, Cat. # AB6046); mouse anti-alpha-tubulin (Santa Cruz Biotechnology, Dallas, TX, USA, Cat #. SC-8035); donkey anti-goat antibody conjugated to Alexa 594 (Invitrogen, Cat. # A-11058); goat anti-mouse antibody conjugated to Alexa 488 (Invitrogen, Cat. # A-11001); goat anti-human IgG-PE (Invitrogen, Cat. # PA1-86978).
Validation	Therapeutic antibodies (Herceptin and Kadcyla) were obtained from clinical preparations at The Ottawa Hospital. All other commercial antibodies were validated on their respective company websites.

Eukaryotic cell lines

Policy information about cell lines

Cell line source(s)	786-0 (human renal carcinoma), GM38 (normal human fibroblasts), T47D (human mammary epithelial), SK-OV3 (human ovarian), MCF-7 (human mammary adenocarcinoma), 4T1 (mouse mammary epithelial) and Vero (African Green Monkey kidney, CCL-81) cells were obtained from the American Type Culture Collection (ATCC). JIMT-1 (human herceptin-resistant breast cancer, ACC589) cells were obtained from the Leibniz Institute DSMZ-German Collection of Microorganisms. 4T1.2 and 4T1.2-HER2 were obtained from the laboratory of Dr. Michael Kershaw (Peter MacCallum Cancer Centre, Victoria, Australia).
Authentication	Cell lines were banked shortly after purchase. Once thawed, cell lines were validated morphologically and used within 3-10 passages since thaw.
Mycoplasma contamination	All cell lines were routinely tested for mycoplasma contamination by Hoechst staining and PCR (Diamed, Mississauga, Ontario, Catalogue # ABMG238).

Commonly misidentified lines (See ICLAC register)

N/A

Animals and other organisms

Policy information about studies involving animals; ARRIVE guidelines recommended for reporting animal research

Laboratory animals	Six to eight week old female CD-1 nude mice (Crl:CD1-Foxn1nu; Charles River Laboratories, Wilmington, Massachusetts, USA, Strain code # 086).
Wild animals	The study did not involve wild animals.
Field-collected samples	The study did not involve samples collected from the field.
Ethics oversight	In vivo experiments were performed via protocols OHRI-2265 and OHRI-2264 which are in good standing with the Animal Care Committee, and care and treatment of animals was in accordance with the ethical standards of the Canadian Council on Animal Care and with the Animals for Research Act.

Note that full information on the approval of the study protocol must also be provided in the manuscript.

Flow Cytometry

Plots

Confirm that:

- [x] The axis labels state the marker and fluorochrome used (e.g. CD4-FITC).
- [x] The axis scales are clearly visible. Include numbers along axes only for bottom left plot of group (a 'group' is an analysis of identical markers).
- [x] All plots are contour plots with outliers or pseudocolor plots.
- [x] A numerical value for number of cells or percentage (with statistics) is provided.

Methodology

Sample preparation	1 x 106 cells were resuspended in 200 µl of FACS buffer (0.5 % BSA-PBS) and transferred to round-bottom 96-well plates and stained with Fixable Viability Dye 510 (BD Horizon, San Jose, California, USA, Cat. # 564406) at 1:1000 for 30 minutes at 4 0C in the dark. Cells were then pelleted at 1500 rpm, 5 minutes, at 4 0C, washed with FACS buffer, then stained with trastuzumab at 1:1000 in FACS buffer (final concentration 21 µg/ml) for 60 minutes at 4 0C in the dark. Cells were then pelleted, washed, and stained with goat anti-human IgG-PE (Invitrogen, Cat. # PA1-86978) at 1:100, for 60 minutes at 4 0C in the dark. Cells were then washed, pelleted, and resuspended in 1 % PFA-PBS and stored overnight at 4 0C. Immediately prior to acquisition, samples were filtered (BioDesign Inc., Carmel, New York, USA, N50R CellMicroSieves, 50 µm pore size) then subjected to flow cytometry using a BD LSRFortessa Flow Cytometer (BD Horizons). Data were analyzed using FlowJo v10.6 software. Unstained controls were prepared in parallel, and PE-treated beads were used for compensation and gating.
Instrument	LSRFortessa Flow Cytometer (BD Horizons)
Software	Data were analyzed using FlowJo v10.6 software.
Cell population abundance	N/A
Gating strategy	Cells were first gated based on size and granularity (FSC-A x SSC-A), singlets were gated based on FSC-A x FSC-H. Then live cells (fixable viability dye APC-Cy7 negative) were assessed for Herceptin-PE-A positivity. A figure is provided in Supplementary Information (Supplementary Figure 2).

- [x] Tick this box to confirm that a figure exemplifying the gating strategy is provided in the Supplementary Information.
